# Increased excitatory synapse size in hippocampal place cells compared to silent cells

**DOI:** 10.1073/pnas.2505322122

**Published:** 2025-06-05

**Authors:** Judit Heredi, Gaspar Olah, Mate Sumegi, Istvan Paul Lukacs, Mohammad Aldahabi, Balázs B. Újfalussy, Judit K. Makara, Zoltan Nusser

**Affiliations:** ^a^Laboratory of Cellular Neurophysiology, Hungarian Research Network Institute of Experimental Medicine, Budapest 1083, Hungary; ^b^Laboratory of Biological Computation, Hungarian Research Network Institute of Experimental Medicine, Budapest 1083, Hungary; ^c^Laboratory of Neuronal Signaling, Hungarian Research Network Institute of Experimental Medicine, Budapest 1083, Hungary

**Keywords:** hippocampus, place cells, synaptic plasticity, patch-clamp, imaging

## Abstract

Neuronal activity in the hippocampus creates a cognitive map of space that is essential for navigation. In any given environment, a fraction of hippocampal pyramidal cells (PCs) is active at specific locations (place cells), others are sparsely active without spatial tuning, and a significant proportion of the PCs is entirely silent. The mechanisms underlying the vastly different activities of PCs in the rodent hippocampal CA1 area are unknown. Here, we measured the in vivo activity of CA1 PCs using two-photon [Ca^2+^] imaging in head-restrained mice during navigation in a virtual corridor and then performed in vitro patch-clamp recording to probe their intrinsic electrical properties and anatomical investigation to characterize their input synapses. The active and passive electrical properties of PCs were similar between PCs with different prior in vivo activities. Perisomatic inhibitory synapse density was also comparable among PCs. The average dendritic spine density and spine head area did not correlate with the mean in vivo activity of PCs, but the size of the spines of place cells was significantly larger compared to that of silent cells. Our results are consistent with excitatory synaptic plasticity as a major mechanism underlying spatially tuned activity of place cells in hippocampal networks.

## Significance

Environment-specific neuronal activity in the hippocampus supports spatial navigation. A substantial fraction of pyramidal cells (PCs) is active whereas other neurons remain silent in a given environment across multiple days, suggesting that the allocation of neurons to a representation is a nonrandom process. Here, we report that PCs with different in vivo activities have similar electrical properties, inhibitory and excitatory synapse densities. However, our data revealed that the size of spines is significantly larger in place cells compared to silent cells. Our results are consistent with excitatory synaptic plasticity as a major mechanism underlying the different activities of hippocampal PCs in vivo.

The hippocampus plays an essential role in spatial navigation by creating distinct internal representations of different environments, based on environment-specific combinations of active principal neurons. Studies in rodents have long established that in any given environment ~20 to 40% of hippocampal CA1 pyramidal cells (CA1PCs) fire action potentials (APs) at specific locations [place cells exhibiting place fields (PFs)], another ~10 to 30% of the CA1PCs display sparse, apparently spatially untuned activity, and in every environment around half of the CA1PCs remain silent (1–6). Whereas the proportion of active cells and place cells has been shown to be relatively stable across environments and days, recent longitudinal recordings tracking the same neurons in well-learned environments revealed gradual evolution of the neuronal representation, such that only a minority of CA1PCs retained consistently similar activities and tuning profiles from day to day (4, 7, 8). A fundamental question for understanding the mechanisms generating hippocampal spatial maps is to clarify the factors that determine the participation of PCs in a given representation.

Previous studies, conducted in large or multiple environments and over prolonged periods indicated that the probability that a CA1PC exhibits spatially tuned activity is not random: place field (PF) propensity follows a highly skewed gamma-Poisson distribution such that the majority of PCs have consistently low propensity to have PF (5, 9, 10). The relatively large fraction of cells with low or no activity in any environment is thought to be advantageous because it ensures sparse coding (11). These observations suggest that PF propensity is an intrinsic and relatively stable feature of CA1PCs that may arise due to genetic and/or developmental programming shaping neuronal excitability. However, the cellular/synaptic/molecular mechanisms underlying this property are not well understood.

It has also been demonstrated that strong electrical or optical excitation of CA1PCs, presumably eliciting large, prolonged depolarization in dendrites that induces synaptic plasticity, can efficiently and rapidly convert silent CA1PCs into place cells (6, 12–14), indicating that any presumed intrinsic mechanism producing silent or sparsely active PCs cannot be strong enough to prevent fast PF formation. Thus, we hypothesize that the widely different activities and PF propensities of CA1PCs are governed by a delicate balance between both persistent and flexible, intrinsic, and synaptic processes.

Here, we aimed to address this hypothesis by performing multiday in vivo two-photon (2P) [Ca^2+^] imaging of identified neurons in the dorsal hippocampal CA1 area while mice navigated in a long virtual corridor. This was followed by post hoc electrophysiological examination of their intrinsic electrical properties in acute brain slices. We have also performed morphological analysis of the density and strength of excitatory and perisomatic inhibitory synapses of in vivo imaged CA1PCs. Surprisingly, we found a lack of significant correlations between the intrinsic electrical properties, perisomatic inhibitory and excitatory synapse densities of CA1PCs and their in vivo activities. The mean spine head area, a measure of excitatory synapse strength, also showed no correlation with the mean in vivo activity of the PCs. However, the size of spine heads was significantly larger in place cells compared to silent cells. These results are consistent with excitatory synaptic plasticity as a key mechanism for the spatially tuned activity of place cells in hippocampal networks.

## Results

### In Vivo 2P [Ca^2+^] Imaging of Hippocampal CA1PCs During Navigation in a Virtual Environment.

**Fig. 1.**
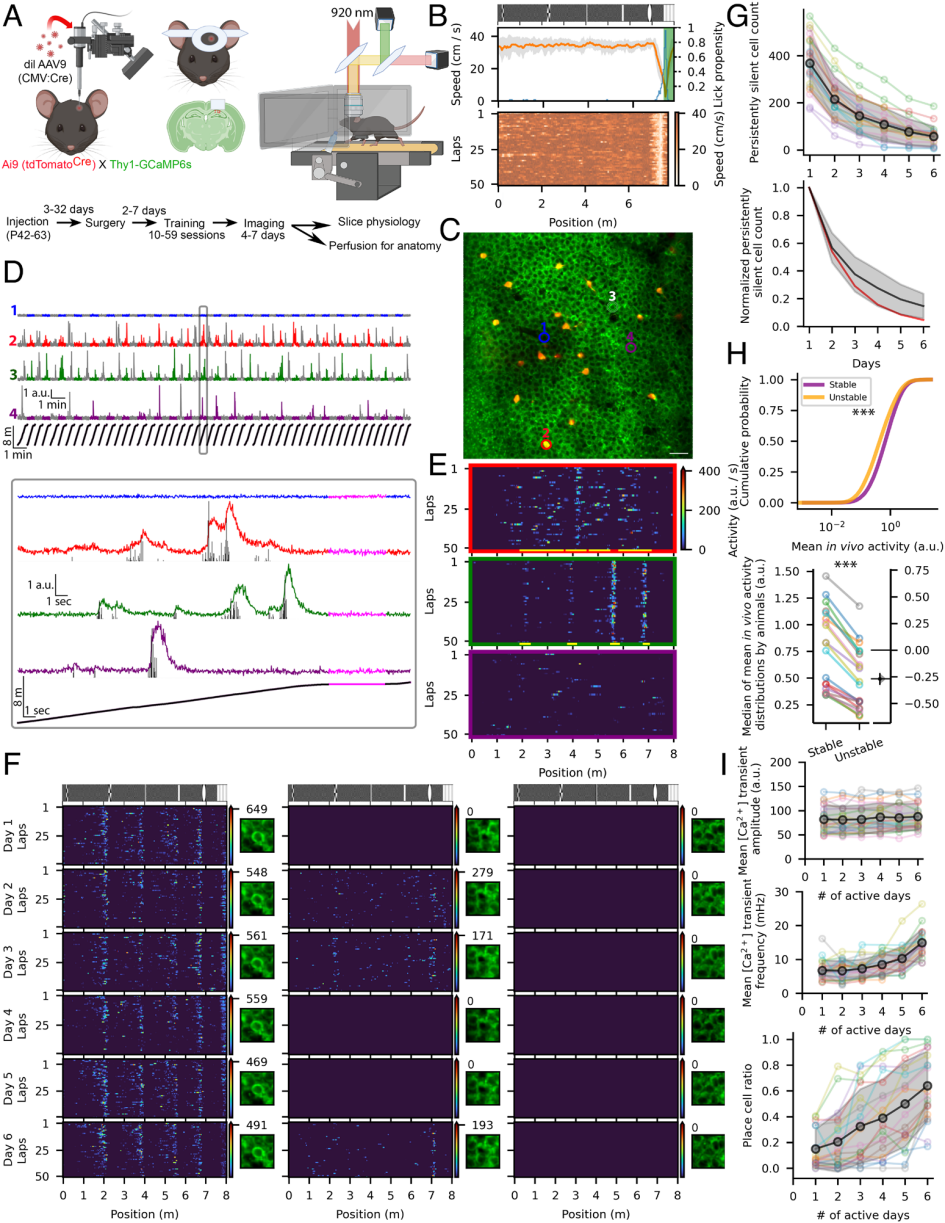
Multiday in vivo 2P [Ca^2+^] imaging from awake mice during spatial navigation in a virtual corridor. (*A*) Experimental timeline. The dorsal hippocampal CA1 area of double transgenic mice was injected with diluted Cre-recombinase containing adeno-associated virus (AAV) to achieve sparse tdTomato labeling for post hoc identification of imaged cells. The craniotomy and the optical cannula insertion were made on the left hemisphere above the dorsal CA1 region. After habituation and training the animals performed a navigational task in an 8-m-long virtual corridor while the CA1 region was imaged with a 2P microscope. (*B*) Behavioral pattern of a representative well-trained animal. The wall pattern of the virtual corridor is shown on the *Top*. Running speed (orange: mean; SD: gray) decreased and lick propensity (blue) increased before the animal reached the reward zone (green area). *Lower* panel shows the lap-by-lap running speed of the animal. (*C*) Representative motion corrected 2P mean image of the CA1 area (mean of 1,000 frames). (Scale bar: 80 µm.) Numbered and color-coded cells are shown in panels *D* and *E*. (*D*) Representative fluorescent [Ca^2+^] traces. The colored traces show the activity of the four cells labeled in *C*. The colored and gray segments represent odd and even laps in the virtual environment. The position of the animal is shown at the bottom with black. (*Bottom*) Fluorescent signals of the four cells with the deconvolved spike activity (black bars) during a single lap. The period labeled with magenta was discarded from further analysis due to the slow running speed of the animal. (*E*) Raster plots show the deconvolved activity of the example cells as a function of the spatial location within the corridor. Significant PFs are indicated by yellow lines. (*F*) Activity of three CA1 PCs in six consecutive days in the same virtual environment. The activity of one cell (*Left*) was space-modulated and was remarkably stable for 6 d. The cell in the middle switched between silent and spatially modulated active states. The cell on the right did not show any detectable activity for 6 d. Images on the right show the recorded cells (*Center*) on each day and the numbers show the maximum activity of the cells on the given day (max values of the color bar). (*G*) Persistently silent cells across 6-d of imaging. (*Top*) Number of persistently silent cells in each imaging session. Each animal is represented with a different color (n = 20). (*Bottom*) Mean counts of persistently silent cells from 20 animals, normalized to their first day’s count (black line, gray shading: SD). The red line represents the theoretical normalized persistently silent cell count, calculated from the mean unsilencing rate (0.54) measured from all consecutive session pairs. (*H*, *Upper*) cumulative probability of the mean activity of persistently active cells (active in all 6 d; purple, n = 30,217) and cells that were silent at least in 1 d (orange, n = 41,147; Kolmogorov–Smirnov test: *P* < 0.001, mean calculated from active sessions only). (*Lower*) The medians of the mean activity distributions of persistently active cells in each animal were significantly higher than those of cells with at least one silent day (paired *t* test: *P* = 1.83*10^−09^). Each animal is represented with a different color. The black dot on the right shows the measured difference and the whiskers represent the 95% CI for the bootstrapped effect size (gray distribution). (*I*) Mean [Ca^2+^] transient amplitude (*Top*) and frequency (*Middle*), and place cell ratio (*Bottom*) plotted as a function of the number of days a cell was active within the 6-d imaging series. CA1PCs with at least one significant PF in at least one session were considered place cells. There is a much larger proportion of place cells among those PCs that were active for 5 to 6 d compared to those that are only active in 1 to 2 d. Black traces: mean, gray shadows: ± SD.

To characterize the activity of identified CA1PCs during spatial navigation over multiple days, we sparsely labeled CA1 neurons with tdTomato by injecting diluted Cre-recombinase-expressing AAVs into the dorsal hippocampus of double transgenic mice in which GCaMP6s is expressed in all nerve cells and the expression of tdTomato is Cre-dependent (Fig. 1*A*). An imaging window was created by implanting a stainless-steel optical cannula above the left dorsal CA1 area (*Materials and Methods*). Animals were then trained to run for water reward in an ~8-m-long linear virtual corridor decorated with six distinct, sparsely spaced visual landmarks on a low-contrast random background pattern, the last of which indicated a reward zone (RZ, Fig. 1 *B*, *Top*). Completion of the lap was followed by teleportation to the start of the track. Well-trained mice ran with a relatively constant speed of ~30 to 40 cm/s through the corridor but slowed down and showed a large increase in lick propensity just before entering the RZ, indicating reward anticipation (Fig. 1 *B*, *Middle* and *Bottom*). Cre-containing AAVs were titrated to achieve a handful of scattered red cells in an imaging field of view (FOV) containing ~1,000 GCaMP6s-expressing CA1PCs (Fig. 1*C*), allowing reliable repositioning of the FOV from day to day as well as post hoc identification of the tdTomato-expressing neurons.

2P [Ca^2+^] imaging was performed in well-trained mice for ~30 min (>50 laps per session) for several days. CA1PCs displayed widely variable somatic activities within a session. In each session, approximately 50% of the cells (50.1 ± 10.8%, n = 120 sessions from 20 mice) did not display any detectable Ca^2+^ activity during the whole session, including running and stop periods: i.e., they were silent (Fig. 1*D*, cell 1). Other PCs exhibited [Ca^2+^] transients during the session with variable rates (Fig. 1*D*, cells 2 to 4). As expected, the position vs. lap raster plots of activity revealed robust spatial modulation of activity in many of the active cells (Fig. 1*E*). Some cells had a single PF, whereas others had multiple (from 2 to 6) PFs, within the virtual corridor (for determination of PFs, see *Materials and Methods*). Place fields of the CA1PCs tiled the entire corridor with increased PF densities around the visual landmarks (*SI Appendix*, Fig. S1*A*).

Our multiday imaging approach allowed us to monitor each PC’s activity for 6 d, revealing substantial day-to-day changes in the activity and the spatial tuning of PCs, resulting in a drift in the neuronal representation of the same virtual corridor (Fig. 1*F* and *SI Appendix*, Fig. S2), similar to that found in freely moving animals (4). Although the neuronal representation of the environment drifted, the proportion of active cells, place cells, and silent cells remained constant throughout days (*SI Appendix*, Fig. S1*E*). While the fraction of silent cells remained stable across days, their identity dynamically changed from day to day, and the number of cells remaining silent for multiple days decreased near-exponentially (Fig. 1*G*). However, the proportion of persistently silent cells deviated from the theoretical exponential decay that was calculated from the day-to-day unsilencing rates measured from all consecutive session pairs (Fig. 1 *G*, *Bottom*), which is inconsistent with a random and independent recruitment of neurons in the active pool and suggests that a specific fraction of the cells could remain persistently silent for a long period. The mean day-to-day activity of those cells that were active throughout the entire 6-d imaging period was significantly higher than that of cells, which were silent at least in one out of the 6 d (Fig. 1*H*). This increased activity was the consequence of higher frequency but similar Ca^2+^ event amplitude (Fig. 1*I*), suggesting that the difference cannot be explained by better signal-to-noise ratio in the more active cells. The proportion of place cells showed a steep positive correlation with the number of days the cells were active during the 6-d imaging series (Fig. 1 *I*, *Bottom*). Finally, we examined whether the expression of Cre-recombinase and the consequent production of tdTomato affected the activity of CA1PCs, and found that the overall activity, the frequency of [Ca^2+^] transients and the proportion of place cells were not significantly different between tdTomato-expressing and nonexpressing cells, only the amplitude of the events was slightly but significantly smaller (*SI Appendix*, Fig. S1*C*). These data taken together demonstrate that many features of hippocampal CA1PC activity that have been described in freely moving rodents are recaptured in our optical recordings from head-restrained mice running in a virtual corridor.

### Similar Active and Passive Electrical Properties of CA1PCs with Different In Vivo Activities.

Can the variability in the in vivo activity of CA1PCs be related to their intrinsic electrical properties? To address this question, we investigated the electrical properties of CA1PCs with known in vivo activity profiles using whole-cell patch-clamp recordings in ex vivo acute slices.

**Fig. 2.**
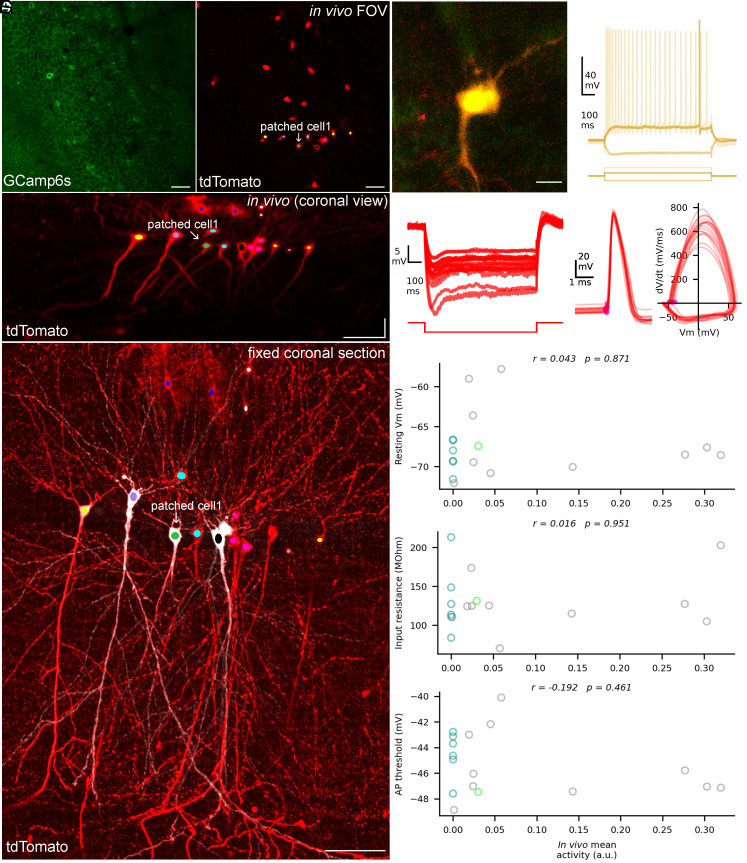
Active and passive electrical properties of in vivo imaged CA1PCs. (*A*) GCamp6s (*Left*) and tdTomato (*Right*) signal in the in vivo FOV. (*B*) Maximum intensity projection (MIP) image of the in vivo z-stack rotated by 90˚ (virtual coronal view). (*C*) MIP image of a coronal brain section chemically fixed after in vivo imaging and in vitro patch-clamp recording. The section was post hoc immunolabeled for TdTomato (red) and biocytin was visualized with Abberior 635P–streptavidin (pseudocolored to gray). Identified PCs are color-coded in panels *A* and *B*. (*D*) A representative 2P image of a tdTomato-expressing PC in a coronal acute slice of the dorsal hippocampal CA1 region (labeled as “patched cell1” on *A*–*C*). (*E*) Firing pattern upon depolarizing current injection of the cell shown in panel *D*. (*F*) Membrane voltage responses to a hyperpolarizing current step (−100 pA; *Left*), APs (*Middle*), and AP phase plots (*Right*) of 17 identified tdTomato-expressing cells that were previously imaged in vivo. (*G*) The resting membrane potential, the input resistance, and the AP threshold of the cells do not show significant correlations with the mean in vivo activity of the cells. The mean in vivo activity refers to the activity of PCs on the last day just before preparation of acute in vitro slices. The green symbol represents the cell shown in *A*–*D*. Silent cells are shown with cyan and active nonplace cells with gray symbols. Spearman correlation coefficient (r) and the *P*-value are shown in the plots. (Scale bars: 50 µm in *A*–*C*, 25 µm in *D*.)

After the last in vivo 2P [Ca^2+^] imaging session, high-resolution Z stacks of the FOV were acquired, followed by preparation of coronal acute slices from the hemisphere containing the imaged dorsal hippocampus. The in vivo imaged area was approximately localized in the in vitro acute slices based on the density and distribution of tdTomato-expressing cells. Next, as many tdTomato positive cells were whole-cell recorded as possible to maximize the likelihood of recording from cells that had been imaged in vivo. Each whole-cell recorded PC was intracellularly filled with biocytin and after the in vitro recordings the slices were chemically fixed and biocytin and tdTomato were visualized with Abberior 635P–streptavidin and anti-tdTomato primary and Cy3-conjugated secondary antibodies, respectively, allowing post hoc 3D alignment and identification of the cells (Fig. 2 *A*–*C*). Throughout the study, we recorded from one hundred and four tdTomato-expressing cells (n = 25 mice), out of which 17 cells were in the in vivo imaging FOV and imaging plane and therefore could be identified (Fig. 2). In addition, 31 tdTomato nonexpressing cells were also recorded as controls (*SI Appendix*, Fig. S3). Active and passive electrical properties of the cells were determined from membrane-voltage responses obtained in response to de- and hyperpolarizing direct current (DC) current injections of different amplitudes (Fig. 2 *E* and *F*). The cell-to-cell variabilities in resting membrane potential (−67.5 ± 4.1 mV), input resistance (129.8 ± 37.2 MΩ), AP threshold (−45.2 ± 2.4 mV) among the post hoc identified, functionally imaged cells (n = 17; see below), were similar to those observed in a much larger population of randomly selected tdTomato-expressing and nonexpressing cells (*SI Appendix*, Fig. S3). We did not find a significant correlation between the resting membrane potential, the input resistance, and the AP threshold vs. the in vivo mean activity of the cells recorded on the last day of in vivo imaging (Fig. 2*G*). We also calculated the average in vivo activity of these cells across all 6 d of imaging, which showed no significant correlations with these electrical properties (*SI Appendix*, Fig. S4).

### Perisomatic Inhibitory Synapse Density Is Similar Among CA1PCs with Different In Vivo Activities.

**Fig. 3.**
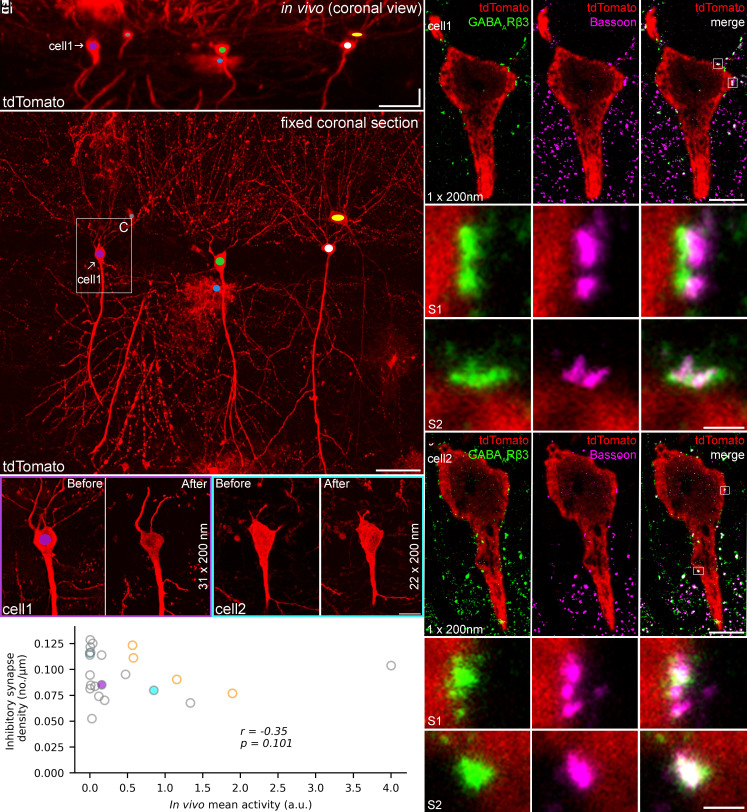
Similar perisomatic inhibitory synapse density on CA1PCs with different mean in vivo activities. (*A*) MIP image of an in vivo z-stack following a 90˚ rotation (virtual coronal view). (*B*) MIP image of a coronal brain section following transcardial perfusion fixation and immunolabeling for tdTomato. The color-coded tdTomato positive cells were identified in panel *A*. The white box indicates the enlarged area in *C*. (*C*) Purple frame: MIP image of the identified, low in vivo activity cell labeled in *A* and *B* (cell1). The cell was digitally reconstructed from thirty-one 200 nm thick sections after resectioning (*Right*). Cyan frame: MIP image of an identified cell (cell2) showing higher in vivo activity and the same cell is shown after reconstruction following resectioning into 200 nm (*Right*). (*D*) Image of cell1 in a single 200 nm thin section immunoreacted sequentially for GABA_A_ receptor β3 subunit (GABA_A_Rβ3) and bassoon and visualized with STED microscopy. White boxes indicate the enlarged areas in *E*. (*E*) Inhibitory synapses of cell1 identified by the colocalization of bassoon and GABA_A_Rβ3 immunosignal. Note the close apposition of the presynaptic (bassoon) and postsynaptic (GABA_A_Rβ3) immunosignals on the merged STED images (*Right*). (*F*) Same as *D* for cell2. (*G*) Same as *E* for cell2. (*H*) Inhibitory synapse density as a function of mean in vivo activity on the last imaging day. Each symbol represents an individual cell (n = 23). Purple and cyan filled symbols indicate the corresponding color-coded frames (cell1 and cell2) shown in *C*. Place cells are represented by orange symbols, active nonplace cells are shown with gray and a silent cell with dark cyan. Spearman correlation r and *P* values are shown in the figure. (Scale bars: 50 µm in A and *B*, 10 µm in *C*, 5 µm in *D* and *F*, 500 nm in *E* and *G*.)

Because we found no robust difference in the intrinsic electrical properties between silent and active cells, we turned our attention to perisomatic inhibition of CA1PCs with different in vivo activities. Inhibition of somatic firing is most effective when GABAergic synapses are localized close to the site of AP generation (perisomatic inhibition). Therefore, we focused on synapses targeting the somata and proximal dendrites of in vivo imaged and identified CA1PCs (Fig. 3). To molecularly identify GABAergic synapses, we performed double immunolocalization of the presynaptic structural protein bassoon and the postsynaptic GABA_A_ receptor β3 subunit (GABA_A_Rβ3). Localization of synaptic proteins is prone to errors when the reaction is done in thick tissue (tens of µm), therefore we performed postembedding immunolocalization on 200 nm thick resin-embedded sections (15) obtained from 120 µm thick sections of the imaged dorsal hippocampus. This requires dehydration, resin embedding, and resectioning of the hippocampus containing the in vivo imaged and post hoc identified tdTomato-expressing PCs (Fig. 3 *A*–*C*). Many bassoon immunolabeled puncta were present in the stratum radiatum, which did not contain GABA_A_Rβ3 immunoreactivity (Fig. 3 *D* and *F*). However, the GABA_A_Rβ3 positive puncta around CA1PC somata and proximal dendrites often colocalized with bassoon immunoreactivity (Fig. 3 *D*–*G*). When the two immunosignals were analyzed at Stimulated emission depletion (STED) resolution, they partially overlapped and the GABA_A_Rβ3 immunosignal was closer to the postsynaptic cell when the synapse was cut perpendicularly (Fig. 3 *E*, *Top*). Overlapping GABA_A_Rβ3/bassoon puncta were considered GABAergic synapses and their densities were determined on the perisomatic region of 23 in vivo functionally characterized PCs. No significant correlation was found between the perisomatic GABAergic synapse density and the mean in vivo activity of the PCs measured either on the last imaging day (Fig. 3*H*) or as the average of activities recorded on the last four to nine imaging days (*SI Appendix*, Fig. S5*A*).

### Place Cells Have Larger Spines Compared to Silent PCs.

**Fig. 4.**
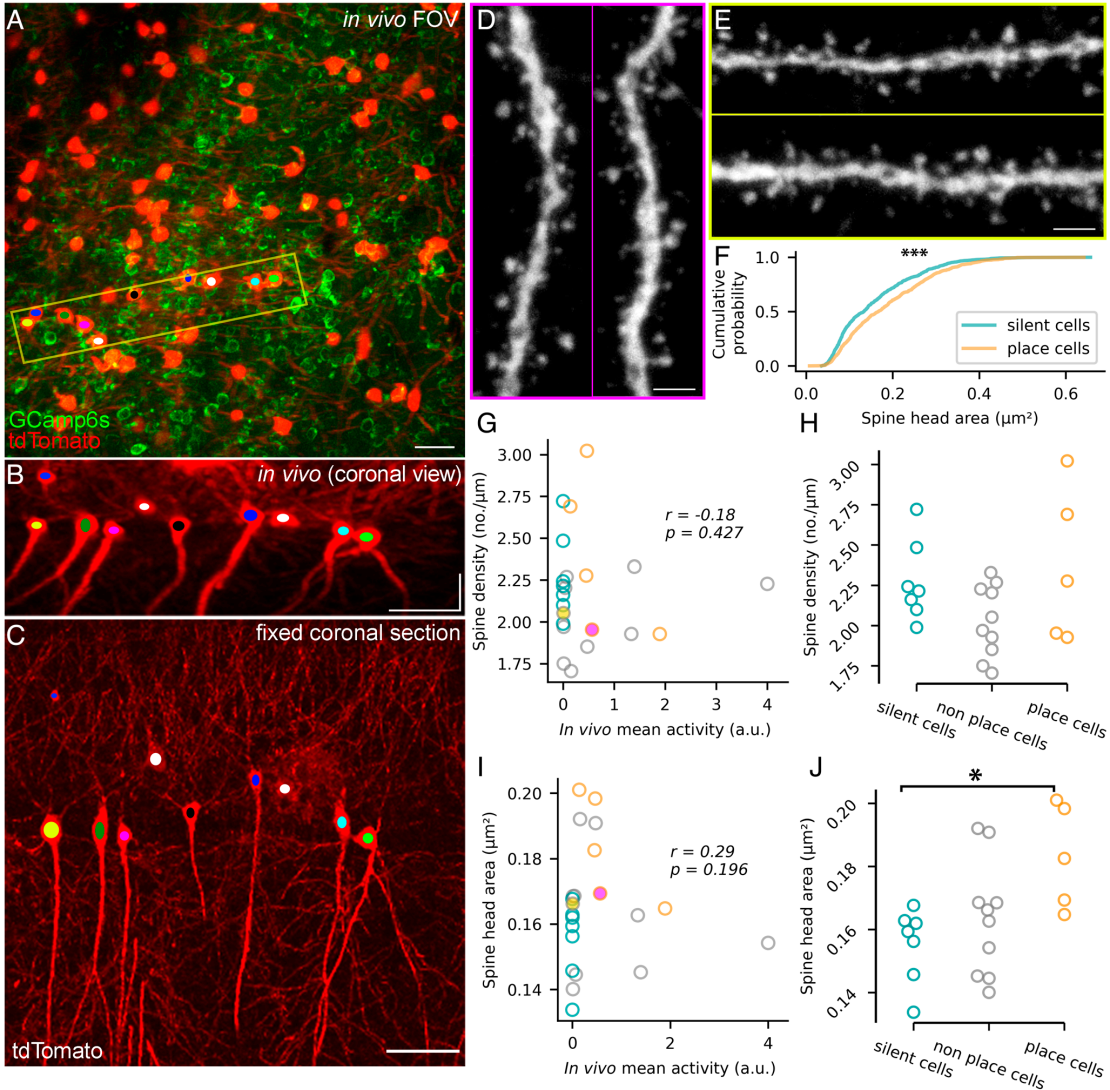
Increased spine head size in place cells compared to silent cells. (*A*) GCamp6s (green) and tdTomato (red) signal in an in vivo FOV. (*B*) MIP image of the in vivo z-stack following a 90˚ rotation (virtual coronal view) of the area shown in panel *A* with a yellow box. (*C*) MIP image of a coronal brain section chemically fixed following in vivo imaging and post hoc immunolabeled for tdTomato. The tdTomato+ cells can be unequivocally identified in the in vivo Z-stack image. Identified PCs are color coded as in *A* and *B*. (*D*) Confocal images of two dendritic segments of the tdTomato+ cell marked with magenta in *C*. (*E*) Confocal images of two dendrites of a cell marked with yellow in *C*. (*F*) Cumulative probability plot of spine head areas of silent (n = 858) and place (n = 632) cells. The two distributions are significantly different (Kolmogorov–Smirnov test, *P* = 6.8*10^−7^). (*G*) Spine density as a function of in vivo mean activity on the last imaging day. Each symbol represents an individual PC (n = 22). Dark cyan symbols represent silent cells, gray symbols show active nonplace cells and orange symbols indicate place cells. The yellow and purple filled symbols represent the two cells shown in panels *A*–*E*. Spearman correlation coefficient (r) and the *P*-values are shown in the plot. (*H*) No significant difference was found in spine density values between silent cells, active nonplace cells, and place cells (*P* = 0.181 Kruskal–Wallis test; *P* > 0.32 Dunn’s post hoc tests). (*I*) Same as *G*, but for the mean spine head area. (*J*) Same as *H*, but for the mean spine head area. A significant difference was observed in mean spine head area values between silent and place cells (*P* = 0.026 Kruskal–Wallis test; *P* = 0.022 Dunn’s post hoc tests). The number of cells and color codes are the same as in *G*. (Scale bars: 50 µm in *A*–*C*, 2 µm in *D* and *E*.)

Finally, we turned our attention to excitatory synapses arriving from CA3PCs, which are thought to primarily drive spatially tuned activity of CA1PCs. Schaffer collateral synapses almost exclusively innervate dendritic spines and the majority of the spines contain a single postsynaptic density (16). Thus, the total pool of excitatory synapses of a cell can be approximated from the density of dendritic spines. Because slice preparation could affect spine densities in PCs (17), we used a novel approach on a new cohort of animals (n = 5) for these experiments. tdTomato-expressing PCs were first imaged in vivo and the animals were then transcardially perfused with an aldehyde-containing fixative immediately after imaging to prevent any morphological alteration. The dorsal hippocampus was resectioned and tdTomato was visualized with immunohistochemistry (Fig. 4). Fig. 4*D* shows confocal images of two apical oblique dendritic segments of a place cell and Fig. 4*E* illustrates those of a sparsely active cell, indicating no apparent difference in the overall morphology and spine density. Quantitative analysis of a total of 22 in vivo imaged and post hoc identified PCs (~10 segments per cell, n = 22 cells) revealed a lack of correlation between spine density and the mean in vivo activity of the cells measured on the last day of imaging (Fig. 4*G*). A similar lack of correlation was found when the activity of the cells was averaged from the last four to six imaging days (*SI Appendix*, Fig. S5*B*). When the mean spine densities were compared in silent cells, active nonplace cells, and place cells, no significant difference was found (Fig. 4*H*; *P* = 0.181 Kruskal–Wallis test).

A previous study from our laboratory demonstrated that the spine head volume and its two-dimensional projected head area show a strong positive correlation with the size of the postsynaptic density (18). Thus, to approximate synaptic strength, we measured spine head areas in the selected dendritic segments (~10 segments per cell, n = 22 cells) and correlated them with the in vivo activity of the parent cells. The mean spine head area showed no significant correlation with the mean in vivo activity as measured either on the last imaging day (Fig. 4*I*) or as the average of the last four to six imaging days (*SI Appendix*, Fig. S5*C*). We then calculated the mean spine head area for every cell and found a significant difference between silent vs. place cells (*P* = 0.026 Kruskal–Wallis test, *P* = 0.022 Dunn’s post hoc test), but not between silent vs. active nonplace cells (*P* = 1 Dunn’s post hoc test) and not between active nonplace cells vs. place cells (*P* = 0.128 Dunn’s post hoc test Fig. 4*J*). Finally, the comparison of the distributions of spine head areas pooled together from all silent and place cells revealed a significant rightward shift for place cells (Kolmogorov–Smirnov test, *P* = 6.8*10^−7^, Fig. 4*F*). Altogether, these results indicate that spines of place cells are larger than those of silent cells.

## Discussion

In the present study, we aimed to determine the mechanisms underlying the widely different activities of hippocampal CA1PCs in mice navigating in a virtual corridor. We opted for a complex approach, based on multiday in vivo 2P [Ca^2+^] imaging of neuronal activity in head-restrained mice performing a virtual navigation/reward-learning task, and subsequent ex vivo patch-clamp electrophysiology and post hoc morphological analysis of identified CA1PCs. Our results revealed that neither the electrical properties nor the overall density of excitatory and perisomatic inhibitory input synapses on PCs show a significant correlation with the in vivo activity of the cells. Whereas the average spine head area (a proxy of excitatory synapse strength) also showed no significant correlation with the overall in vivo activity of CA1PCs, place cells had larger spines than silent cells. Our results are consistent with excitatory synaptic plasticity as a major mechanism underlying the different tuning properties of CA1 PCs in vivo.

It is well known that the rodent hippocampus represents different environments via environment- and location-specific activity of PCs. Among PCs, some exhibit spatially untuned firing, whereas others display spatially tuned activities, and a significant fraction of PCs is completely silent (1–6). When the animal explores a completely novel environment, a new hippocampal representation rapidly emerges, whereas less drastic alterations in an environment often result in partial or rate remapping (19). In addition to remapping due to changes in the environment, the neuronal representation also undergoes slow evolution from day to day even in an unchanged familiar environment, a process called representational drift (4, 7, 8). Both reorganization processes (remapping and drift) result in robust changes in the activity and/or spatial tuning of individual PCs, including conversion of active cells to silent and vice versa (2, 20, 21). One possibility is that the selection of active cells is a random process, giving each PC an equal chance to become part of or fall out from a representation. Based on a random model, each PC should have an equal chance to possess single or multiple PFs in a new environment or when an environment is extended, which is an often-occurring natural behavior of wild rodents as they explore larger and larger space around their nest in search of food. This hypothesis was tested and refuted using recordings in large physical or virtual environments, which revealed that CA1PCs have widely different PF propensities, the distribution of which is well described by a highly skewed gamma-Poisson process (5, 9, 10). The propensity of PFs of individual PCs is preserved in different environments and over a long period of time, providing strong evidence against a random process (9). Here, we followed the activity of CA1PCs over six imaging days in a highly familiar environment. Similar to previous studies, we observed substantial day-to-day reorganization of neuronal activity. Furthermore, the proportion of silent cells remaining completely inactive over six imaging days was higher than expected from a random unsilencing of previously silent cells, consistent with the above conclusions that place cell recruitment into a new representation is mediated via a nonrandom selection process.

What might be the cellular mechanisms underlying the different PF propensities and the actual activity of PCs in a given representation? A possible explanation is that the intrinsic electrical properties of the cells are different, and they determine the probability with which a cell will be recruited into a novel representation. Indeed, PF propensity of CA1PCs measured by in vivo [Ca^2+^] imaging in cue-rich virtual environments correlated well with the spontaneous activity of the cells in total darkness, an apparently sensory cue-less condition (9). Similarly, cells with spontaneously high activity rates were more likely to become engram cells in a hippocampal memory test (22). Epsztein et al. (23) directly tested the above hypothesis by performing in vivo patch-clamp recordings in anesthetized and freely moving rodents and found a lower AP threshold and a higher burst firing propensity in future place cells compared to future silent cells, while somatic membrane potential and input resistance were not significantly different. Our results using post hoc in vitro patch-clamp recordings of previously in vivo imaged CA1PCs are at odds with these findings. We found no significant correlations between the input resistance, resting membrane potential, and the AP threshold vs. the in vivo activity of the cells. When we calculated the average day-to-day in vivo activity of these cells from multiple imaging days, it showed significant correlation with neither of these properties. Thus, our data argue against intrinsic somatic excitability serving as a major cause of the different in vivo activities of CA1PCs in a familiar environment.

Another potential mechanism explaining the extended silence of some hippocampal PCs could lie in their inhibitory inputs. Here, we estimated the perisomatic inhibitory synapse densities, which did not correlate with the overall in vivo activity of the neurons. Nevertheless, we cannot rule out that potential differences in inhibitory synaptic strength might contribute to the vastly different activities of CA1PCs (24).

As another major candidate mechanism, excitatory synaptic plasticity has long been proposed to play a role in the generation and/or consolidation of place cells. Although PFs appear in novel environments even under the blockade of N-Methyl-D-aspartic acid (NMDA) receptors indicating that place cells can receive sufficiently strong spatially tuned input ab ovo (25), NMDA receptors have been shown to contribute to PF stabilization (26). Interestingly, several recent studies (6, 12–14, 27–30) demonstrated that spontaneous occurrence of a single large and prolonged somatic depolarization, exhibiting signatures of dendritic plateau potentials (6, 13), is sufficient to convert silent cells to place cells, an effect that can also be induced artificially by strong somatic depolarization at any random location of the environment. Several properties of the newly formed PFs (e.g., enhanced ramp-like depolarization with increased V_m_ variance) were consistent with synaptic potentiation (6, 13). Experiments performed in acute brain slices (13) confirmed that postsynaptically evoked dendritic plateau potentials can potentiate coactivated excitatory synapses in stratum radiatum with a synaptic plasticity mechanism (termed Behavioral Time Scale Synaptic Plasticity: BTSP) exhibiting an asymmetric, seconds-long temporal kernel, a window much wider than that described for spike-timing-dependent synaptic plasticity (STDP; tens of milliseconds) (31–33). Furthermore, BTSP was not accompanied by noticeable changes in somatic excitability in vivo (12). The fact that any silent cell could be almost instantly and persistently transformed into a place cell with an experimentally induced synaptic plasticity mechanism argues against fundamentally distinct intrinsic electrical properties of silent vs. place cells. In contrast to the above studies, Cohen et al. (34), using similar recording methods, found that the majority of newly formed PFs in a novel environment displayed no signatures of BTSP, but the strength of a weak initial subthreshold spatially tuned depolarization increased by PF formation. This implies that other plasticity mechanisms, e.g., STDP-like (31–33) or local dendritic long-term plasticity induction mechanisms (35–37) might also take place during PF formation. Thus, it is still an open question whether (or under which conditions) BTSP is the dominant synaptic plasticity mechanism underlying PF formation or other forms of plasticity forms also contribute (38).

According to our analysis, the most prominent difference between place cells vs. silent cells was that the apical oblique dendrites of place cells harbored spines with larger size, a parameter that closely correlates with synapse strength. This finding is consistent with long-term potentiation of Schaffer collateral excitatory synapses contributing to PF formation and/or stabilization in CA1PCs and suggests that the strength of spatially tuned input could also be the major determinant for setting the activity of place cells across days (i.e., the stronger the input-mediated depolarization, the more reliably the cell will be reactivated every day). The question arises as to which synaptic plasticity mechanism was responsible for creating such differences and whether there are intrinsic genetic differences among CA1PCs regarding their ability to undergo robust synaptic plasticity. Although the molecular mechanisms of BTSP have not been clarified, they may be similar to those of the well-characterized classical STDP at these synapses, manifesting in an increased α-Amino-3-hydroxy-5-methyl-4-isoxazolepropionic acid receptor number, postsynaptic density size, and spine head volume. Thus, the above question can only be addressed experimentally if molecular pathways underlying BTSP are uncovered, possibly allowing selective and specific molecular perturbation of distinct plasticity mechanisms. Furthermore, future studies will be required to explore the synaptic mechanisms underlying de novo formation of PFs in completely novel environments. In summary, our results provide evidence supporting the notion that long-term synaptic plasticity processes play a prominent role in shaping the continuously evolving ensemble code that underlies navigation and spatial memory in behaviorally relevant environments.

## Materials and Methods

### Animals.

Male (n = 13) and female (n = 21) Ai9xGP4.3 double-transgenic mice were used. Animals were housed in the vivarium of the Institute of Experimental Medicine in a normal 12 h/12 h light/dark cycle and had access to water and food ad libitum. All the experiments were carried out according to the regulations of the Hungarian Act of Animal Care and Experimentation 40/2013 (II.14) and were reviewed and approved by the Animal Committee of the Institute of Experimental Medicine, Budapest.

### Virus Injection.

Mice were anesthetized with an intraperitoneal injection of CP-ketamine (ketamine hydrochloride, 62.5 mg/kg), CP-Xylazine (xylazine, 12.5 mg/kg), and Pipolphen (promethazine hydrochloride, 6.25 mg/kg). For local analgesia, Naropine (ropivacaine hydrochloride 50 to 100 μL, 2 mg/mL) was injected subcutaneously. The eyes of the animal were covered with Opticorn A ointment. The head was fixed in a stereotaxic frame and a ~0.5 mm diameter craniotomy was made above the virus injection site. 100 nL pENN.AAV.hSyn.Cre.WPRE.hGH (3.3 × 10^10^ GC/mL, Addgene #105553-AAV9) was injected at AP: −2, DV: −1.1, ML: 1.8 coordinates in the left hemisphere with a 2.3 nL/s flow rate in four bouts separated by 1 min pauses. For postsurgery analgesia and anti-inflammatory treatment, Bupaq (buprenorphine hydrochloride 0.1 mg/kg) and Rimadyl (carprofen 25 mg/kg) were subcutaneously administered. During the 2 d of postoperative care, the animal’s drinking water was supplemented with 35 ng/mL of carprofen.

### Surgery.

3 to 32 d after the virus injection the animals were anesthetized with Isoflurane (1.5% in carbogen, flow rate 0.8 L/min). A 3 mm diameter craniotomy was drilled over the virus injection site and the cortex above the hippocampus was gently aspirated. A 3 mm diameter 1.7 mm long stainless-steel cannula with a glass coverslip attached to the bottom was inserted and cemented in place together with a custom 3D printed titanium head bar. During the 2 d of postoperative care, the animal’s drinking water was supplemented with 35 ng/mL of carprofen.

### Behavioral Training.

Water bottles were removed from the home cage 24 h before training started (2 to 7 d after optic cannula surgery). Training and in vivo 2P imaging were carried out in the middle of dark hours. During training days, the animals had no access to drinking water; they could drink only 5% sucrose solution as a reward. If an animal could not keep at least 85% of its starting weight, 30 min after the end of the training/imaging sessions free access to water was granted for 10 min. On the first day of the training, the animals were head-fixed in a 2 m length version of the virtual environment (Luigs & Neumann with a custom Labview and PyOgre software) and trained to run on a treadmill by giving small drops of reward whenever the animal was running. After animals learned to run on the belt and were only given rewards in the reward zone if they licked a lickport at the end of the virtual corridor, the length of the corridor was gradually increased to 8 m by adding new segments to the beginning of the virtual maze. Animals learned to run at least 50 laps in the 8 m long virtual environment after 10 to 59 training sessions (median = 20.5 sessions).

### In Vivo 2P [Ca^2+^] Imaging.

The CA1 region of the left dorsal hippocampus of well-trained animals was imaged during a ~50-lap session. (Power: 70 mW at the sample, λ: 920 nm, 30 Hz sampling rate, 512 × 505 frame size, with the Femto-2D Dual 2P microscope and Nikon LWD 16× NA = 0.80 objective lens). To facilitate the post hoc identification of the in vivo imaged PCs, animals were anesthetized with isoflurane (1.5%) after the last behavioral imaging session and a z image-stack was acquired. After in vivo imaging, the animals were killed for either in vitro electrophysiology or were transcardially perfused for morphological investigations.

### In Vitro Electrophysiology.

Briefly, the animals were anesthetized with isoflurane and then decapitated. The brain was removed from the skull in ice-cold cutting solution containing (in mM): sucrose, 205.2; KCl, 2.5; NaHCO_3_, 26; CaCl_2_, 0.5; MgCl_2_, 5; NaH_2_PO_4_, 1.25; and glucose, 10, bubbled with 95% O_2_ and 5% CO_2_, and 250 to 300 µm thick coronal slices were cut from the dorsal hippocampus with a vibratome (VT1200S, Leica, Wetzlar, Germany) and placed in a submerged chamber in ACSF containing (in mM): NaCl, 126; KCl, 2.5; NaHCO_3_, 26; CaCl_2_, 2; MgCl_2_, 2; NaH_2_PO_4_, 1.25; glucose, 10 saturated with 95% O_2_ and 5% CO_2_ (pH = 7.2 to 7.4) at 36 °C for 30 min, which was then gradually cooled down to 22 to 24 °C. Recordings were carried out in the same ACSF at 32 to 33 °C for up to 6 h after slicing. Patch pipettes were pulled (Zeitz Universal Puller; Zeitz-Instrumente Vertriebs, Munich, Germany) from thick-walled borosilicate glass capillaries with an inner filament (1.5 mm outer diameter, 0.86 mm inner diameter; Sutter Instruments, Novato, CA). Pipette resistance was 5 to 6 MΩ when filled with the intracellular solution containing (in mM): K-gluconate, 130; KCl, 5; MgCl_2_, 2; EGTA, 0.05; creatine phosphate, 10; HEPES, 10; ATP, 2; GTP, 1; biocytin, 7; (pH = 7.3; 290 to 300 mOsm). Recordings were obtained using either a Multiclamp 700A or a Multiclamp 700B amplifier (Molecular Devices, CA). Voltage signals were filtered at 6 kHz (Bessel filter) and digitized at 50 kHz with DigiData 1550A AD converter (Molecular Devices, CA). Data were collected using pClamp10_7 software (Molecular Devices, CA). tdTomato-expressing cells were targeted for recordings under an epifluorescent or a 2P microscope. The series resistance of the pipette was bridge-balanced throughout the experiments.

### Tissue Preparation.

After in vitro recordings slices were placed in a fixative containing 4% formaldehyde (FA) and 0.2% picric acid in 0.1 M PB phosphate buffer (pH = 7.4) for 12 h at 4 °C. Then they were embedded in agarose (2%) and resectioned at ~120 µm thickness. The biocytin-filled cells were visualized with Abberior 635P-conjugated streptavidin (1:1,000, Abberior) in Tris-buffered saline containing 0.2% Triton X-100.

Animals that were not used for in vitro electrophysiology (n = 5) were deeply anesthetized and were transcardially perfused with a fixative containing 4% FA and 0.2% picric acid in 0.1 M PB (pH = 7.4). The brains were removed and postfixed overnight in the same fixative then 120 µm thick coronal sections were cut from the dorsal hippocampus. To enhance the tdTomato signal, sections were immunoreacted with a rabbit anti-tdTomato primary antibody (1:1,000, Frontier) and a Cy3 coupled secondary anti-rabbit antibody (1:200, Jackson ImmunoResearch).

### Cell Identification and Dendrite Analysis.

To post hoc identify in vivo imaged PCs, tdTomato signals were matched on the in vivo 2P z-stack of the FOV (rotated 90°) and on the z-stack of the coronal slices from in vitro slices or from sections of perfusion fixed brains. Post hoc identified cells whose dendrites were analyzed were reconstructed with Neurolucida software (MicroBrightfield).

Z-stacks of apical dendritic segments were imaged using an Abberior Instruments FacilityLine STED microscope (60× 1.4 NA objective on an Olympus BX63 microscope, Abberior Instruments GmbH, Göttingen, Germany) and STED and confocal images were simultaneously obtained. The spine density of the dendrites was analyzed with Neurolucida, while the spine head area was measured on maximum intensity projection images with the ImageJ plugin SpineJ (39).

### Postembedding Sequential Immunofluorescent Labeling.

To evaluate perisomatic inhibition on the in vivo imaged and post hoc identified cells, slices were first dehydrated and flat-embedded in epoxy resin (Durcupan). Tissue sections containing the identified tdTomato-expressing cells were re-embedded, and 200 nm thick serial sections were cut and mounted on adhesive Superfrost Ultra plus histological slides. Etching the resin, antigen retrieval, immunolabeling, and elution of the staining were carried out as reported previously (15). Antigen retrieval was modified as follows: samples were placed in the same retrieval solution, but sodium dodecyl sulfate was omitted and the solution was heated in a microwave oven at maximum power (850 W) for 3 × 5 min.

For identification of GABAergic synapses, the following primary and secondary antibodies were used: rabbit anti-VGAT (1:200, Synaptic Systems), rabbit anti-GABA_A_Rβ3 [1:200, gift from V. Sieghart, Medical University of Vienna; (40)], rabbit anti-Bassoon (1:200, Synaptic Systems) and goat anti-rabbit IgGs coupled to Abberior 635P (1:200, Abberior) in three consecutive staining and elution steps. Images of the perisomatic area of identified PCs were taken using a STED microscope. GABA_A_Rβ3+/Bassoon+ synapses were counted on every second 200 nm thick serial section and their numbers were normalized to the perimeter of the perisomatic membrane length.

### Analysis of In Vivo Ca^2+^ Imaging Data.

Motion correction, region of interest (ROI) detection, fluorescent trace extraction, and spike deconvolution were performed with the software Suite2p (41). The Suite2p output files were further analyzed by custom-made Python scripts. To obtain a conservative estimate of the activity of each neuron, spikes corresponding to small amplitude events in the fluorescent trace were removed. Briefly, we estimated the noise amplitude of the fluorescent signal both locally (around the spike) and globally (using the entire trace) and included only spikes where the amplitude of the corresponding Ca^2+^ event was significantly larger than the noise. Specifically, local maxima of the neuropil subtracted fluorescence (Fc) were identified between each spike and the following nine frames. Each Fc maximum was replaced with the mean of three Fc values around the detected local maximum (Fc_max_). Next, we estimated the local linear trend of the baseline by fitting a linear to the Fc values between −30 to −20 frames preceding each event. The local noise SD was estimated by the RMSE of the linear fit, and we used the linear prediction at the −20th frame position as the baseline (Fc_base_). The global noise SD was defined as the SD of the Fc values between 0.5*Fc_med_ – 1.5*Fc_med_, where Fc_med_ is the median of the entire Fc trace. An event was considered valid if its amplitude, Fc_max_ − Fc_base_, was larger than local or the global noise SD. The spike trains noise-filtered this way were used for all further analysis. The mean activity of each neuron was defined as the mean of the inferred spike count across all time bins using the noise-filtered spike trains. Event frequency, amplitude, and the mean activity of the cells were calculated from these traces.

Since Suite2p uses ROI detection based on the correlation between the fluorescent activity of neighboring pixels, it could not find neurons with low activity. Therefore, to identify the inactive neurons we used Cellpose (42) on the mean image output of Sute2p to segment all the cells in the FOV. To connect the Cellpose ROI set to the Suite2p ROI set we calculated the spatial overlap of ROI masks, and we considered the two ROIs coming from the same cell if the overlap between the masks was higher than 75% of the Cellpose mask size. ROIs identified by Cellpose with no corresponding Suite2p mask were considered silent cells for the analysis in Fig. 1 *G*–*I*.

To follow the cells’ identity through multiple days we registered the mean images and ROI sets from single days. First, we quantified the silent cells in the FOV. Then we registered the mean images of the first and second recording days, and we calculated the spatial overlap of Cellpose ROIs. In this manner, we were able to connect those ROIs that were visible on the mean image regardless of their activity. Then we repeated the same procedure on all consecutive days. Only those cells were analyzed which had a Cellpose ROI on every recording day. To distinguish between [Ca^2+^] transients originating from the tdTomato positive cells from neighboring/out of focus PCs, we implemented the method of Gauthier et al. (43). Briefly, first we segmented the ROIs on the red channel with Cellpose. Then the fluorescence (the mean of the pixel values within a ROI) was extracted for every frame. The raw fluorescent signal was smoothed for event detection (Gaussian filter, sigma: 2 frames). The putative [Ca^2+^] events were detected if the signal was higher than the median +2 SD of the trace. The spatial footprint was then calculated for every putative [Ca^2+^] event as in ref. 43 and was correlated with the ROI’s shape. The events showing larger than 0.5 spatial correlation values were accepted for further analysis.

### Place Cell Determination.

First, space bins where the average speed of the mouse was below 4 cm/s were excluded from further analysis. Next, the odd–even tuning curve correlation coefficient for each ROI was determined by computing the Pearson correlation coefficient from the tuning curves (average Suite2p spks values in each space bin) calculated only from odd or even laps. ROIs with smaller than 0.25 odd–even lap correlation were excluded from further place cell analysis. Next, spatial information content of each ROI was determined [global spatial information of spatial information (SI); (44)]. For testing significance, the data were randomized by circularly rotating the Suite2p spks output in the time dimension, then SI content was recalculated. This bootstrapping step was repeated 1,500 times and a cell was considered a putative place cell if its global SI score was larger than the 99th percentile of the shuffled SI values. In the next step, the tuning curve of each place cell was used for determining the putative PFs. Peaks of the tuning curve were defined by scipy.signal.find_peaks function (height = 6) (45). If the local minimum between two adjacent peaks was higher than half of the larger peak amplitude, the smaller peak was discarded. The base width and base position of the selected peaks were determined with the scipy.signal.peak_widths function (rel_height = 0.93) using the output of scipy.signal.peak_prominences function (45). Peak base start and end values were replaced by the closest space bin integer values covering the whole base. Adjacent or overlapping peak bases were unified into a single peak. Next, local SI content for each peak was calculated. First, the base area of each peak was extended by 0.5 widths for both directions, and a SI calculation with bootstrapping was performed similarly to the global SI score. In this case, the selected region’s spks data was circularly permuted with a random time shift in each lap. Bootstrap repetition was 1,500 and the significance criterion for PF selection was the 95th percentile of the shuffled distribution. Peaks with significant local SI were only considered PFs if there was at least one event in at least 30% of the laps. Putative place cells with at least one significant PF were considered true place cells for a given session.

### In Vitro Electrophysiology Analysis.

The in vitro electrophysiological data were analyzed with custom Python scripts. The electrophysiological properties of the cells were determined as follows: AP threshold: the voltage where the first time derivative of the voltage signal exceeded a 10 mV/ms threshold; Resting Vm: membrane potential value without current injection; Input resistance: mean of the input resistance values calculated from the amplitudes of the membrane potential change upon different DC current injections that lacked APs divided by the injection current amplitude.

### Statistical Analysis.

We used Spearman correlations for mean in vivo activity vs. cell properties plots. In the case of comparison of two populations we first investigated the normality of the populations with D’Agostino and Pearson’s test. If the populations were normally distributed then we used *t* tests, otherwise we conducted nonparametric tests (e.g., Mann–Whitney *U* test for the spine head area comparison). For comparing the cumulative probabilities Kolmogorov–Smirnov test was used. For comparing the spines densities and spine head areas in three distinct cell groups, we used the Kruskal–Wallis test with Dunn;s post hoc test. We considered the results of the tests significant at *P* value < 0.05. We used bootstrapping to test the significance of the difference between the distributions in Fig. 1*H* and *SI Appendix*, Fig. S3. In these cases, we calculated the CI around the mean difference by resampling the data points (with replacement) n = 5,000 times.

## Supplementary Material

Appendix 01 (PDF)

## Data Availability

Excel files data have been deposited in HUN-REN ARP (hdl:21.15109/ARP/JELBUI) (46).
