# Supplementary material for: Increased excitatory synapse size in hippocampal place cells compared to silent cells
Source: Proc Natl Acad Sci U S A. 2025 Jun 5;122(23):e2505322122. doi: 10.1073/pnas.2505322122 (PMC12167973; doi:10.1073/pnas.2505322122)
Supplement: Supplementary file 1 — Appendix 01 (PDF) [file pnas.2505322122.sapp.pdf]

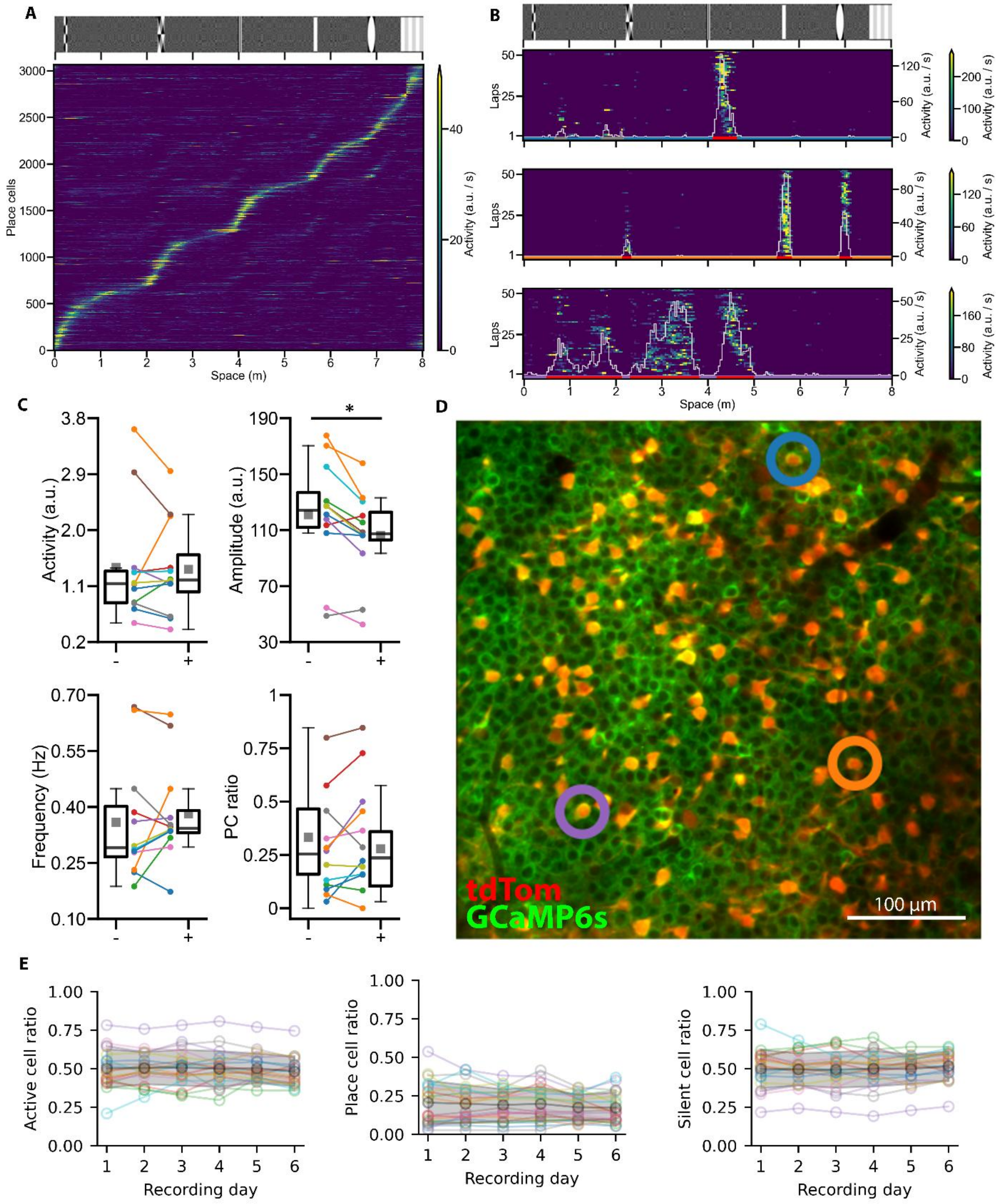

Fig. S1. Characterization of place cell activity in the dorsal hippocampus during virtual navigation. (*A*) Distribution of place fields of CA1 PCs along the virtual corridor. Place cells (pooled from 20 mice) sorted according to the position of their highest activity. Place fields cover the entire virtual corridor with marked enrichments of the place cells around visual landmarks of the maze. (*B-D*) Expression of tdTomato does not alter the activity of CA1 PCs in the dorsal hippocampus. (*B*) Activity raster plots from three tdTomato-expressing PCs (red horizontal bars: significant place fields). (*C*) Comparison of the *in vivo* activity and place cell prevalence between tdTomato-expressing (+) and non-expressing cells (-). Colors represent individual animals (n = 11, p(Activity) = 0.813, p(Amplitude) = 0.004, p(Frequency) = 0.388, p(place cell ratio) = 0.133; two tailed paired t-tests with Bonferroni correction).

Horizontal lines in the box plots: 25th, 50th, and 75th percentiles, rectangle: mean, whiskers: SD. (*D*) Representative microphotograph of the FOV (red: tdTomato, green: GCaMP6s). Color-coded circles indicate the cells shown in *B*. (*E*) Active cell, place cell and silent cell ratios are plotted against consecutive imaging days. The colors represent individual animals. The gray dots show the mean across animals and ±SD is shaded. Only cells recorded for 6 days are included.

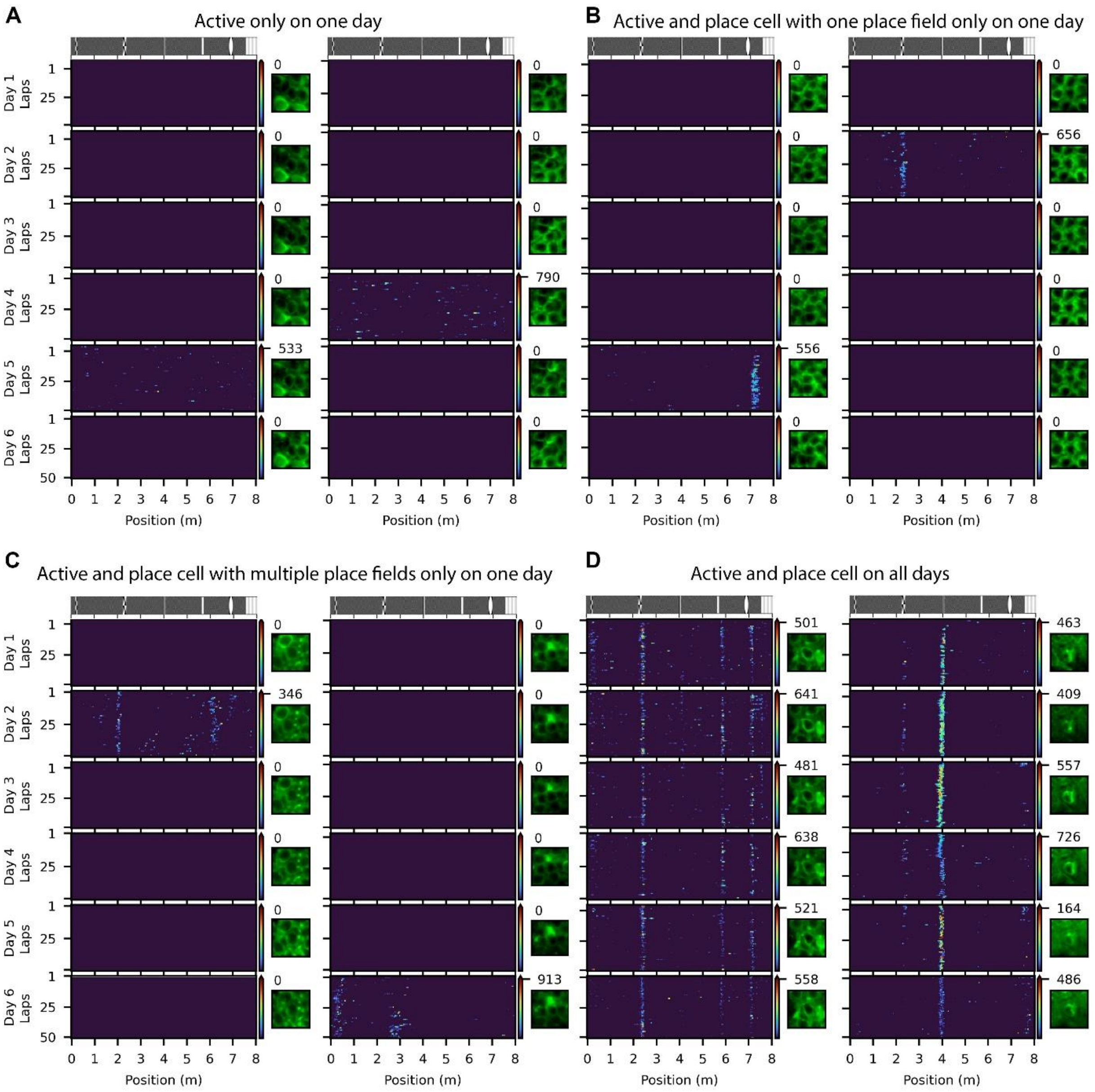

Fig. S2. CA1 PCs with distinct multiday *in vivo* activities. (*A*) Two CA1 PCs showing sparse activity only in one out of the 6 recording days. (*B*) Two PCs were silent for 5 days, but when they were active, their activity was place modulated. (*C*) Same as B, but the cells had multiple place fields. (*D*) Two example cells with reliable place-modulated activity throughout the 6 experimental days. On the right side of the activity raster plots the view of the cells are shown. The relevant cells are always in the center of the image. The color maps of the activity were normalized for every day and the maximal activity of the cells on the given day are shown, similar to Fig1 F.

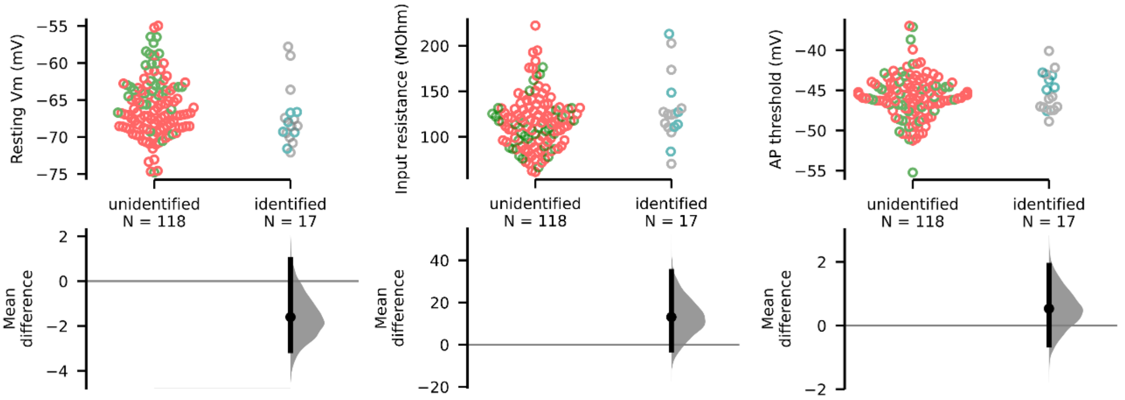

Fig. S3. Active and passive electrical properties of CA1 PCs with known *in vivo* activities are compared to those of randomly recorded PCs. The active and passive electrical properties were measured in acute hippocampal slices using whole-cell recordings. Green symbols represent tdTomato negative PCs (n = 31) that were recorded in the same acute slices. Red symbols represent PCs that expressed tdTomato (n = 87). The unidentified cells were patched in a region of the slice, which was not imaged *in vivo* so they were not exposed to the laser. The identified cells consist of the 17 tdTomato-expressing PCs that are shown in Figure 2 (gray: active non-place cells, cyan: silent cells). Bootstrapped effect sizes (mean difference compared to unidentified cells) are plotted below. The black dot shows the measured difference and the whiskers represent the 95% confidence intervals for the bootstrapped effect size (gray distribution). No significant difference was found between the identified and unidentified CA1 PCs.

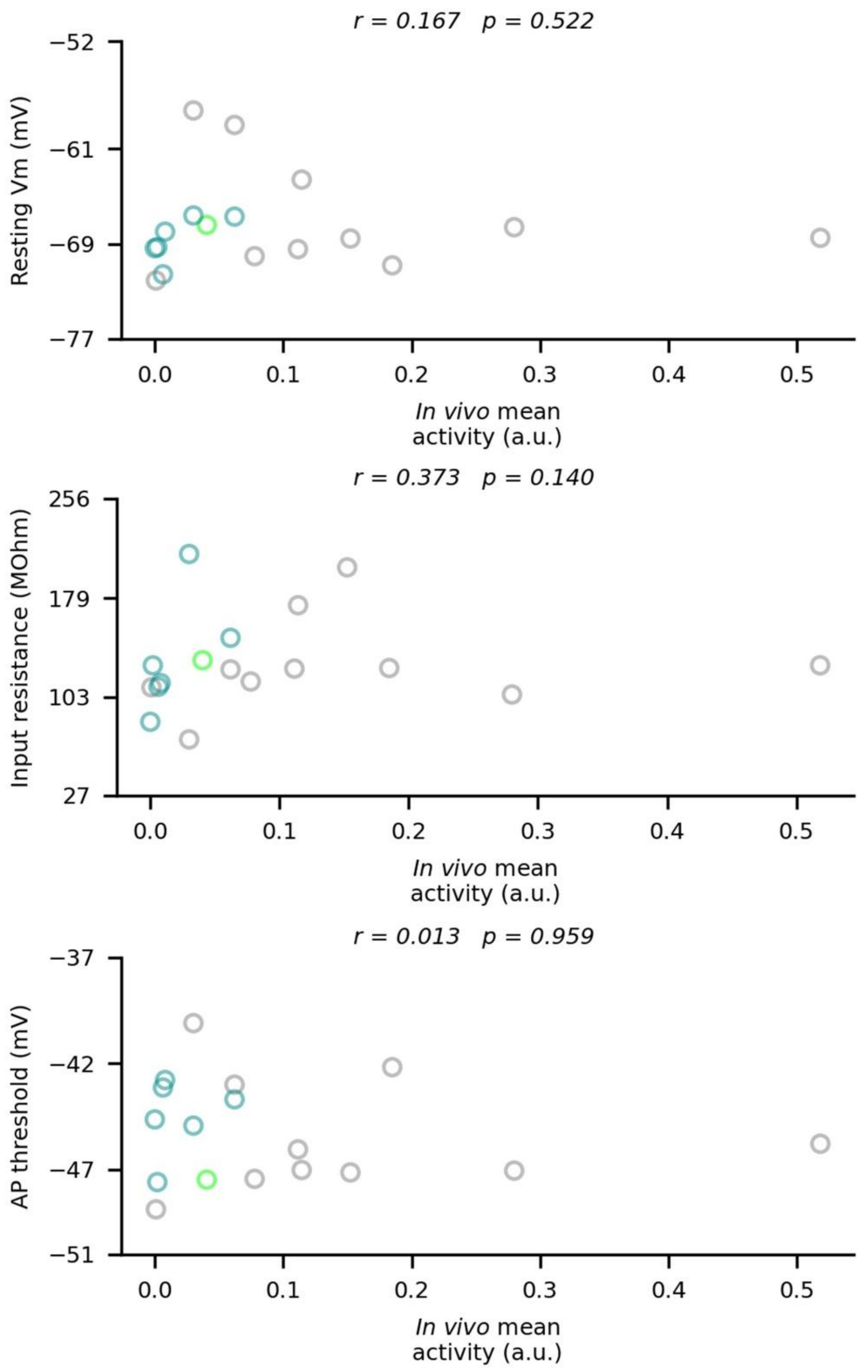

Fig. S4. Correlations of intrinsic electrical properties and multiday mean *in vivo* activity of PCs. Resting membrane potential, input resistance and action potential (AP) threshold do not show significant correlation with the mean *in vivo* activity of CA1 PCs (n = 17) calculated from multiday recordings as the average of the day-by-day activity. Color code same as Fig. 2*G* (dark cyan: cells that were silent on last day, gray: cells that were active, but non-place cells on last day, green: example cell on Fig. 2*C* - *E*).

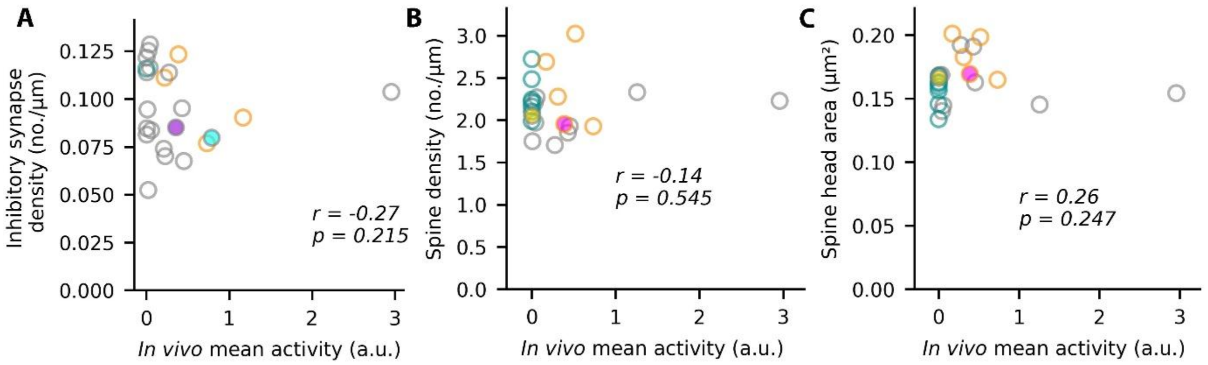

Fig. S5. Correlations between synaptic parameters and multiday *in vivo* activity of CA1 PCs. The inhibitory synapse density (*A*; n = 23 cells), spine density (*B*; n = 22 cells) and spine head area (*C*; n = 22 cells) on CA1 PCs show no significant correlation with the mean *in vivo* activity of PCs calculated from multiday recordings as the mean day-by-day activity. Color code in *A* same as in Fig. 3*H*. Color code in *B* and *C* same as in Fig. 4*F*, *G*.
